# Spatiotemporal Dynamics of Single and Paired Pulse TMS-EEG Responses

**DOI:** 10.1007/s10548-020-00773-6

**Published:** 2020-05-04

**Authors:** Annika A. de Goede, Irene Cumplido-Mayoral, Michel J. A. M. van Putten

**Affiliations:** 1 Department of Clinical Neurophysiology, Technical Medical Centre, University of Twente, P.O. Box 217, Technohal 3385, 7500 AE Enschede, The Netherlands; 2 Biomedical Engineering, Technical Medical Centre, University of Twente, Enschede, The Netherlands; 3 Department of Neurology and Clinical Neurophysiology, Medisch Spectrum Twente, Enschede, The Netherlands

**Keywords:** Transcranial magnetic stimulation, Electroencephalography, TMS-EEG, Spatiotemporal dynamics, Long intracortical inhibition, Stability

## Abstract

For physiological brain function a particular balance between excitation and inhibition is essential. Paired pulse transcranial magnetic stimulation (TMS) can estimate cortical excitability and the relative contribution of inhibitory and excitatory networks. Combining TMS with electroencephalography (EEG) enables additional assessment of the spatiotemporal dynamics of neuronal responses in the stimulated brain. This study aims to evaluate the spatiotemporal dynamics and stability of single and paired pulse TMS-EEG responses, and assess long intracortical inhibition (LICI) at the cortical level. Twenty-five healthy subjects were studied twice, approximately one week apart. Manual coil positioning was applied in sixteen subjects and robot-guided positioning in nine. Both motor cortices were stimulated with 50 single pulses and 50 paired pulses at each of the five interstimulus intervals (ISIs): 100, 150, 200, 250 and 300 ms. To assess stability and LICI, the intraclass correlation coefficient and cluster-based permutation analysis were used. We found great resemblance in the topographical distribution of the characteristic TMS-EEG components for single and paired pulse TMS. Stimulation of the dominant and non-dominant hemisphere resulted in a mirrored spatiotemporal dynamics. No significant effect on the TMS-EEG responses was found for either stimulated hemisphere, time or coil positioning method, indicating the stability of both single and paired pulse TMS-EEG responses. For all ISIs, LICI was characterized by significant suppression of the late N100 and P180 components in the central areas, without affecting the early P30, N45 and P60 components. These observations in healthy subjects can serve as reference values for future neuropsychiatric and pharmacological studies.

**Electronic supplementary material:**

The online version of this article (10.1007/s10548-020-00773-6) contains supplementary material, which is available to authorized users.

## Introduction

A proper balance between excitation and inhibition is essential for normal physiological brain function. Various neuropsychiatric conditions, such as epilepsy, autism and schizophrenia, appear to be related to an imbalance in cortical excitability (Bauer et al. 2014; Bunse et al. 2014; Bolden et al. 2017; Oliveira et al. 2018). Paired pulse transcranial magnetic stimulation (TMS) can be used to assess cortical excitability, as well as to obtain information about the relative contribution of excitatory and inhibitory networks. TMS combined with electromyography (EMG) uses a peripheral motor response as the final readout. Here the interval between the conditioning and test pulse determines whether the conditioning pulse enhances or attenuates the evoked test response in the target muscle compared to an unconditioned muscle response (Valls-Solé et al. 1992; Kujirai et al. 1993; Rossini et al. 2015; Ziemann 2017). For intervals between 50 and 400 ms, the test response is normally reduced where the degree of inhibition varies per interval. This phenomenon is known as long intracortical inhibition (LICI) (Valls-Solé et al. 1992) and is associated with gamma-aminobutyric acid (GABA)-B receptor mediated inhibition (McDonnell et al. 2006; Müller-Dahlhaus et al. 2008).

Combining TMS with electroencephalography (EEG) makes it possible to assess LICI directly at the cortical level (Daskalakis et al. 2008; Fitzgerald et al. 2008). Applying a conditioning pulse 100 ms in advance induces significant suppression of the average TMS evoked potential (TEP), similar to the attenuation of the muscle response (Daskalakis et al. 2008; Fitzgerald et al. 2008, 2009; Farzan et al. 2009, 2010; Garcia Dominguez et al. 2014). More recent studies found a significant decrease of all characteristic TEP components compared to the single pulse TEP (Rogasch et al. 2013, 2015; Premoli et al. 2014b; Opie et al. 2017, 2018), except for an increase of the P70 (Premoli et al. 2014b). The interstimulus interval (ISI) of 100 ms is most commonly studied using TMS-EEG, because it results in strong LICI of the muscle response (Daskalakis et al. 2008; Opie et al. 2017). Measuring LICI at various ISIs could further increase our understanding of the underlying inhibitory mechanisms and assist to characterize well-known TMS-EMG findings with TMS-EEG.

Only one group additionally evaluated an ISI of 150 ms and found a decrease of the N40, N100 and P180, but no significant effect for the P30 component (Opie et al. 2017, 2018). The fact that a significant decrease of the P30 component was only found for ISI 100 ms and not for ISI 150 ms (Opie et al. 2017, 2018), might indicate that the relative contribution of inhibitory mechanisms associated with LICI varies between ISIs. Opie et al. (2017) speculated that LICI at ISI 100 ms might reflect the activation of both pre- and post-synaptic GABA-B receptors, whereas only pre-synaptic GABA-B receptors are activated at ISI 150 ms. However, the same study found no significant differences between both intervals for any of the paired pulse TEP components, suggesting a uniform inhibitory effect irrespective of ISI (Opie et al. 2017). This is supported by the finding of a similar TEP modulation for short intracortical inhibition (SICI) and LICI (Premoli et al. 2018). Knowledge obtained with the TMS-EMG paradigm might therefore not be directly translatable to TMS-EEG outcomes.

Besides the possibility of measuring LICI at the cortical level, TMS-EEG has a spatial resolution of about 10 mm and a millisecond temporal resolution (Ilmoniemi et al. 1997; Fecchio et al. 2017). Applying a single pulse to the motor cortex results in an immediate, localized and strong response at the stimulation site. Within 5–10 ms activation spreads to adjacent ipsilateral motor and premotor areas, followed by the activation of contralateral homologous cortical areas after 20 ms (Ilmoniemi et al. 1997; Komssi et al. 2002). Based on topographical plots, the P30 component is located in central areas, the N45 in contralateral frontal areas, the P70 over the stimulation site, where the N100 and P180 components show a wide distribution over central and centrofrontal areas, respectively (Paus et al. 2001; Bonato et al. 2006; Ferreri et al. 2011; Premoli et al. 2014a). For ISIs 100 and 150 ms, the paired pulse TEP has a similar spatiotemporal dynamics and LICI is expressed at the location of the corresponding TEP component (Premoli et al. 2014b; Opie et al. 2017, 2018).

So far, our knowledge of the paired pulse TEP and LICI is solely based on measurements performed at ISIs 100 and 150 ms, while longer ISIs may also be of interest. For example, previous TMS-EMG studies only found significant differences between drug-naïve epilepsy patients and healthy controls for LICI at ISIs 250 and 300 ms (de Goede et al. 2016). In this study, we use five ISIs between 100 and 300 ms to stimulate both hemispheres in healthy subjects during two sessions one week apart. These findings in healthy subjects not only increase our understanding of TMS-EEG excitability measures, but can also serve as reference values for future neuropsychiatric and pharmacological TMS-EEG studies. Ultimately, the clinical applicability of TMS-EEG, as well as the ability to use our findings as reference values, depend on the stability and repeatability over time of the TEP. To evaluate the stability and repeatability of TMS-EEG outcomes, we investigate the effect of stimulated hemisphere, time and coil positioning method.

We aim to evaluate the spatiotemporal dynamics and stability of the healthy single and paired pulse TEP, and assess LICI directly at the cortical level.

## Materials and Methods

The study protocol (trial ID: NL49854.044.14) was in accordance with the Declaration of Helsinki and approved by our local medical ethics committee (Medisch Spectrum Twente, Enschede, the Netherlands). Furthermore, we followed the guidelines for the use of TMS in clinical practice and research (Rossi et al. 2009). We reported earlier on TMS combined with electromyography (EMG) obtained from the same dataset (de Goede and van Putten 2017; de Goede et al. 2018).

### Subjects

Healthy adults (18 years or older) were included after giving written informed consent. Subjects with contraindications to TMS as described in the ‘Screening Questionnaire before TMS’ (Rossi et al. 2011) were excluded. Subjects were recruited locally by posting flyers at the University of Twente and the Medisch Spectrum Twente. Of the thirty-four inclusions, nine subjects were excluded: one did not complete the first session after not feeling well, one could not be measured a second time within 1–2 weeks due to illness, two could not be stimulated at 120% of the resting motor threshold (rMT) due to a high rMT, and in five it was not possible to stimulate both hemispheres during both sessions due to technical problems or a high rMT. Therefore, data from twenty-five subjects (5 males, mean age 28.2 ± 8.3 years; range 20–51 years, 23 right-handed) was used for further analysis.

Subjects filled out the Dutch Handedness Questionnaire (van Strien 1992; Strien 2003) to determine handedness in order to make a distinction between the dominant and non-dominant hemisphere during analysis.

### TMS Protocol

Subject were instructed to keep their eyes open and head in a fixed position with both hands pronated and relaxed during stimulation. To mask the sound of the TMS pulses, each subject listened to adaptive noise played at a maximum intensity of 95 dB (ter Braack et al. 2013b).

Both motor hotspots of the abductor digiti minimi (ADM) muscle were stimulated using biphasic TMS pulses, with a pulse duration of 400 µs, applied by a Magstim Rapid^2^ stimulator (The Magstim Company Ltd, Whitland, United Kingdom). Stimulation intensity depended on the rMT, which was defined as the minimum intensity needed to induce at least five motor evoked potentials (MEPs), with an amplitude larger than 50 µV, out of ten consecutive pulses. First 50 single pulses were applied, followed by 50 paired pulses at each of the five randomly applied ISIs: 100, 150, 200, 250 and 300 ms. The single pulse intensity, as well as the conditioning and test pulse intensities, were set to 120% rMT. A random interval ranging from 3.5 to 4.5 s was kept between single pulses and pairs of paired pulses.

All subjects underwent this TMS session twice under equal circumstances, including the same measurement set-up, coil positioning method, investigators and time of the day. Both sessions took place approximately one week apart (mean 7.4 days, range 6–15 days).

### Coil Positioning

The 70 mm figure-of-eight air film coil (The Magstim Company Ltd, Whitland, United Kingdom) was placed tangentially at the ADM hotspot, with the handle pointing backwards and laterally at a 45° angle with the midline. In sixteen subjects the coil was positioned and held in place manually by the same investigator, during both TMS sessions. In the other nine subjects, a robot-guided system (Smartmove; ANT Neuro, Enschede, the Netherlands) was used for coil positioning. The position of the subject with respect to the coil was continuously monitored by a Polaris infrared camera system (Northern Digital, Waterloo, Canada), using a headband with four passive reflective markers. A general magnetic resonance image was used to create a head model that was linked to each subject by collecting three landmarks and at least 300 additional points from the scalp with a tracking pointer. Once the ADM hotspot was located manually, it was added to the generated head model. A robotic arm holding the coil was used for positioning at the indicated location and displacements were automatically detected and actively corrected to ensure accurate and stable coil positioning throughout the TMS session.

### EEG Recording and Analysis

During single and paired pulse TMS, the EEG was recorded continuously using either the NeuroCenter EEG or ASA software (Clinical Science Systems, Leiden, the Netherlands and ANT Neuro, Enschede, the Netherlands, respectively), a 64-channel full-band EEG amplifier (TMSi, Oldenzaal, the Netherlands) and a TMS-compatible EEG cap (ANT Neuro, Enschede, the Netherlands). The 64 Ag/AgCl electrodes were positioned using the 10/10 layout, with the ground electrode positioned between electrodes Fpz and Fz. The EEG signal was sampled at 4000 Hz (NeuroCenter EEG software combined with manual coil positioning) or 2048 Hz (ASA software combined with robot-guided coil positioning) and low-pass filtered with an anti-aliasing filter (cut-off frequency 0.2 times the sampling frequency).

The EEG was analyzed in the common average montage and down sampled to 1000 Hz. We excluded disconnected electrodes (no signal > 10% of the recording) and electrodes containing a lot of noise (e.g. eye-blinks or muscle activity). TMS responses were first baseline corrected by subtracting the mean amplitude over a period of 350 to 100 ms before applying the single or conditioning pulse. Trials were defined from 50 ms before till 650 ms after each single or conditioning pulse, resulting in 50 single pulse trials and 50 paired pulse trials per ISI with a length of 700 ms for each electrode. We applied single trial principal component analysis (PCA) using 40 principal components for TMS artifact reduction. It is an effective method to reduce the first large artifact, which results from the magnetic pulse, as well as the second artifact, which is assumed to be caused by muscle activation on the scalp. For a detailed description of the PCA method used, see ter Braack et al. (2013a). We removed the first four principal components, after which trials were filtered between 1 and 45 Hz using a fourth order Butterworth bandpass filter. By taking the average over 50 trials, the single pulse and uncorrected paired pulse TEPs were obtained for each electrode position. All TEPs were analyzed over the entire frequency range between 1 and 45 Hz.

In order to test repeatability of the single and paired pulse TEPs over time, we calculated for each subject the amplitudes of the two most prominent TEP components (N100 and P180) at electrode position Cz. Both these late components showed significant suppression in the central area when assessing LICI at the cortical level, in contrast to the early components. The N100 amplitude was defined as the largest negative value over an interval of 70–130 ms after giving the single or test pulse and the P180 amplitude as the largest positive value between 130 and 220 ms. Based on visual inspection of the TEPs, these intervals were occasionally slightly adapted.

**Fig. 1:**
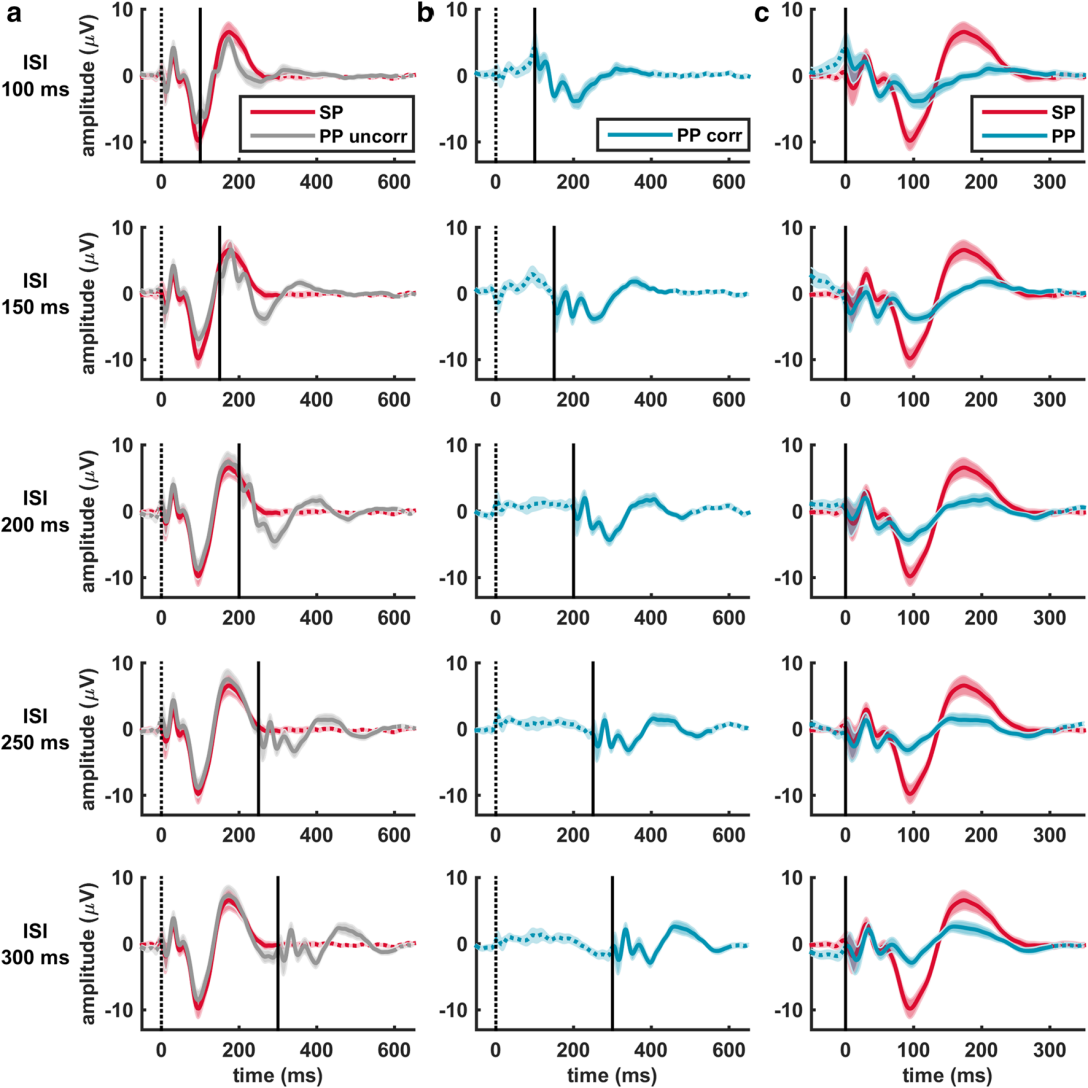
Representation of the paired pulse correction method applied to correct for the influence of late conditioning response components on early test response components. **a** The single pulse TEP (SP in red) is subtracted from the uncorrected paired pulse TEP (PP uncorr in grey) to obtain **b** the corrected paired pulse TEP (PP corr in blue), referred to as paired pulse TEP. **c** Alignment of the single pulse (SP in red) and paired pulse TEP (PP in blue) enables assessment of LICI at the cortical level. Especially the late N100 and P180 components are strongly suppressed for all five ISIs. Each TEP is the average over all subjects (mean ± standard error of the mean (SEM)) at electrode Cz for stimulation of the dominant hemisphere during session 1. The moment of applying the single or conditioned pulse is indicated by the black dotted line and the moment of the test pulse by the black straight line

Interpretation of the paired pulse TEP is not straightforward, due to the fact that, in contrast to the MEP, the TEP is still ongoing when applying the test pulse. Thus, the early components of the response evoked by the test pulse are most likely affected by the late components of the response evoked by the conditioning pulse. Simultaneously, these same late components of the conditioning response are most likely influenced by the induced activity of the test pulse. However, these two interactions are so entangled that they cannot be disconnected (Premoli et al. 2014b). Figure 1 describes the paired pulse correction method we applied to correct for the first interaction: the influence of late conditioning response components on early test response components (Daskalakis et al. 2008; Premoli et al. 2014b). The corrected paired pulse TEP, hereafter referred to as paired pulse TEP, was obtained by subtracting the single pulse TEP from the uncorrected paired pulse TEP. Alignment of the single and test pulse enables direct comparison of the single and paired pulse TEP for the assessment of LICI, see Fig. 1c.

### Statistical Analysis

To assess LICI at the cortical level, the cluster-based permutation analysis (Maris and Oostenveld 2007) was applied as implemented in FieldTrip. This is a statistical method to analyze spatiotemporal dynamics of multidimensional TMS-EEG data, without being affected by the multiple comparison problem. Each ISI was analyzed individually for stimulation of the dominant and non-dominant hemisphere. To compensate for handedness, topographical plots of left-handed subjects were mirrored (e.g. TEPs of electrodes C3 and C4 were exchanged). We used dependent samples *t*-tests to test for differences between the single and paired pulse TEPs indicating LICI. Comparisons were performed for each electrode and each time sample over a period of 300 ms after giving the single or test pulse. Clusters were formed by *t*-values with a *p*-value < 0.05, based on adjacent time samples and neighboring electrodes (n ≥ 2). To determine significance, the summed *t*-value of each cluster was statistically tested against the distribution of clusters obtained by permutation. Using the Monte Carlo method, TEPs were randomly assigned to either the single or paired pulse condition for 1500 times. Clusters found in the original data were considered to be significant if less than 5% of the summed *t*-values obtained by permutation exceeded the original cluster *t*-value, i.e. if *p*-value < 0.05.

Comparable analyses were applied to evaluate stability of the single and paired pulse TEPs. For the effect of stimulated hemisphere, dependent samples *t*-tests were used to test for differences between TEPs measured after stimulating the dominant and non-dominant hemisphere. For this, the topographical plots of non-dominant stimulation were mirrored. For the effect of time, TEPs of both TMS sessions were compared using dependent samples *t*-tests. Finally, for the effect of coil positioning method, independent samples *t*-tests were used to test for differences between TEPs obtained by manual and robot-guided positioning. When no significant differences are found this would point towards a stable measure, although *t*-tests are not the most appropriate method to test for similarities between conditions. In addition, the intraclass correlation coefficient (ICC) was used to estimate the agreement between repeated sessions; model ICC (3,1): two-way mixed single measures, absolute agreement (Shrout and Fleiss 1979). The N100 and P180 amplitudes of all subjects measured during the first session were correlated with all the individual N100 and P180 amplitudes from the second session. For this we combined the N100 and P180 amplitudes of both hemispheres and coil positioning methods.

Matlab (version R2015a, The Mathworks, Natick, MA, USA) was used for the statistical as well as EEG and TEP analysis. We considered a *p*-value < 0.05 to be statistically significant and additionally adjusted for the number of tests performed (Bonferroni corrected with n = 2 for LICI and the effect of stimulated hemisphere and time, and n = 4 for the effect of coil positioning method). Effect sizes were calculated using Cohen’s d, defined as $d= \frac{{\mu }_{1} - {\mu }_{2}}{s}$, with the pooled standard deviation s defined as $s=\sqrt{\frac{\left({n}_{1} - 1\right) * {s}_{1}^{2} + \left({n}_{2} - 1\right) * {s}_{2}^{2}}{{n}_{1} + {n}_{2} - 1}}$. We determined the effect size of significant clusters by taking the mean over all significant electrodes and time samples. Effect sizes above 0.8 or below − 0.8 were considered to be large (Cohen 1988). ICC varies between 0 and 1, where 1 represents perfect repeatability. We considered ICC values above 0.8 as good, values from 0.6 to 0.8 as moderate and values below 0.6 as poor repeatability (Du et al. 2014).

## Results

In sixteen of the twenty-five included subjects the coil was positioned manually, while robot-guided coil positioning was applied in nine. No adverse events happened and all participants tolerated the stimulation protocol well, except for the first excluded subject.

### Spatiotemporal Dynamics of the Single and Paired Pulse TEP

**Fig. 2:**
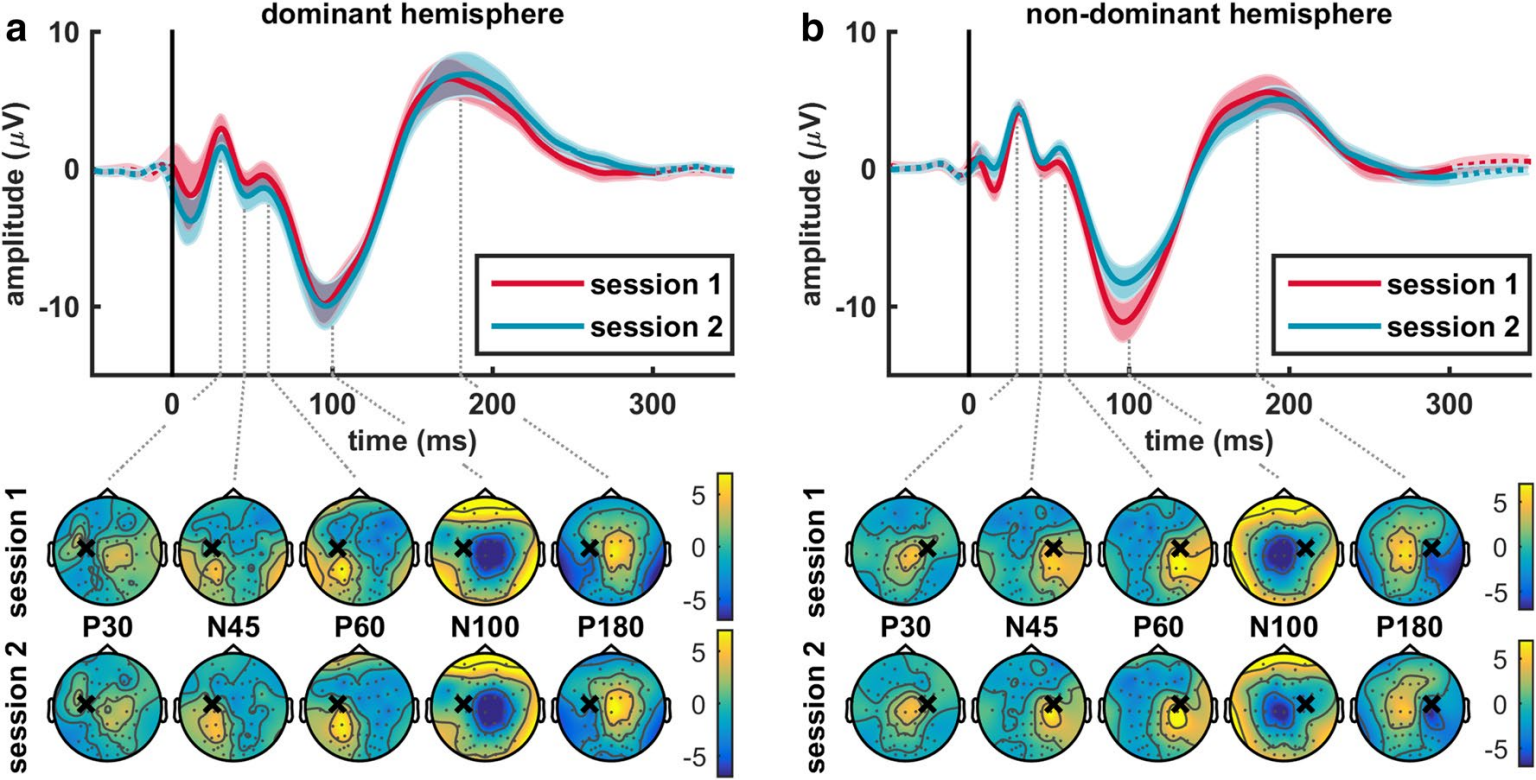
Average single pulse TEP and topographical plots of the characteristic TEP components for both TMS sessions when stimulating the **a** dominant or **b** non-dominant hemisphere. No significant differences were found between the single pulse TEPs of session 1 (in red) and session 2 (in blue), nor between TEPs measured after stimulating the dominant (on the left) and non-dominant (on the right) hemisphere. Each TEP is the average over all subjects (mean ± SEM) at electrode Cz. The topographical plots show the distribution of the P30, N45, P60, N100 and P180 components, where the black cross indicates the stimulation site and the grey dots the 64 electrode positions. Yellow areas indicate a positive amplitude and blue areas a negative amplitude

**Fig. 3:**
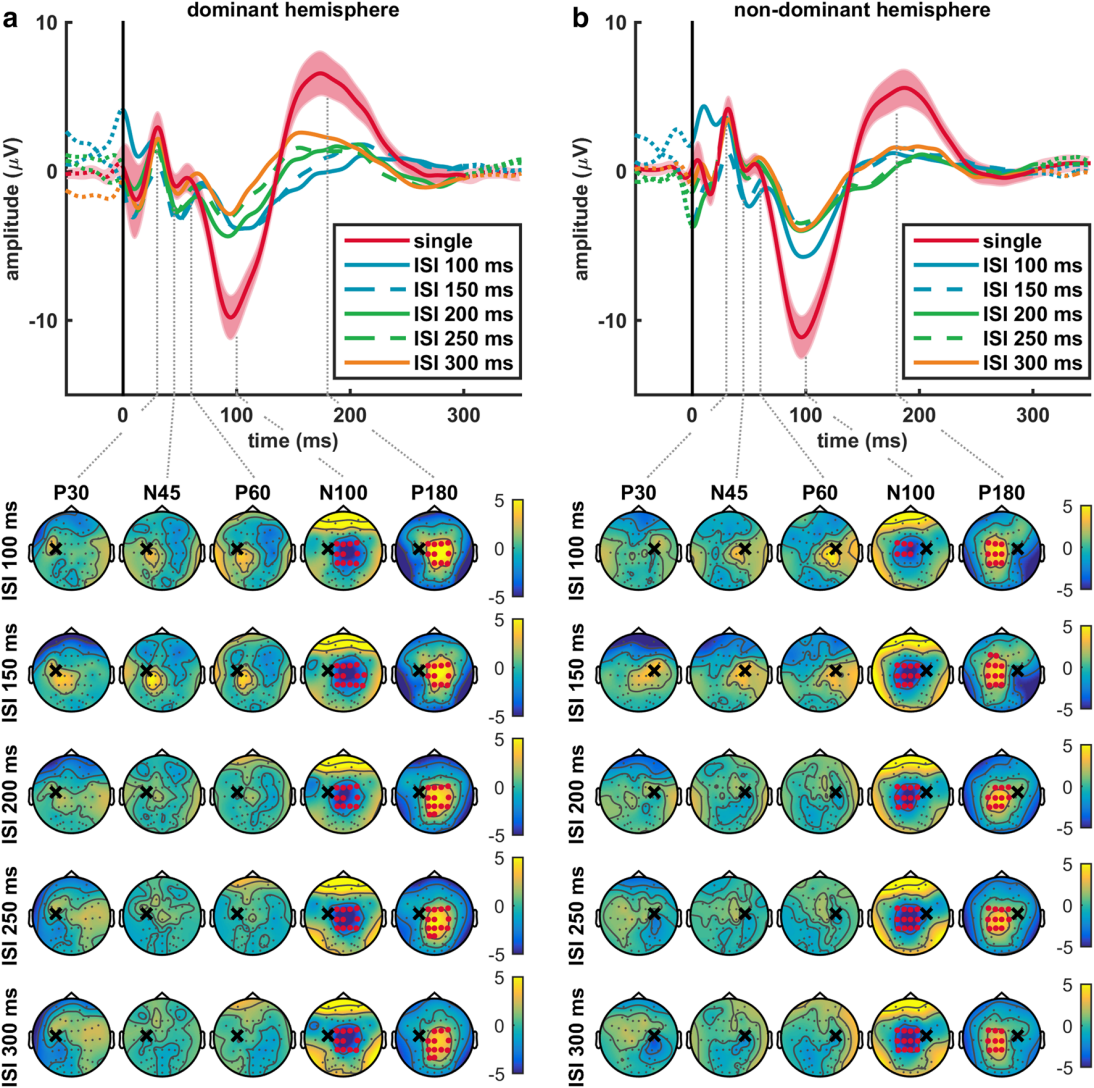
Average single and paired pulse TEPs and topographical LICI plots of the characteristic TEP components when stimulating the **a** dominant or **b** non-dominant hemisphere during session 1. We assessed LICI by comparing the single versus paired pulse TEP. The late N100 and P180 components are strongly suppressed for all five ISIs, while the early P30, N45 and P60 components remained unaffected. Significant negative N100 and positive P180 clusters were found in central areas, corresponding to suppression at the expression site of the late components. The single pulse TEP is the average over all subjects (mean ± SEM) at electrode Cz, while for each paired pulse TEP only the mean is presented. The topographical plots show the distribution of LICI for the P30, N45, P60, N100 and P180 components. The black cross indicates the stimulation site, the grey dots the 64 electrode positions, and the red dots electrodes that show a significant difference between single and paired pulse TEPs. Yellow areas indicate a reduction of positive amplitudes and blue areas a reduction of negative amplitudes

**Fig. 4:**
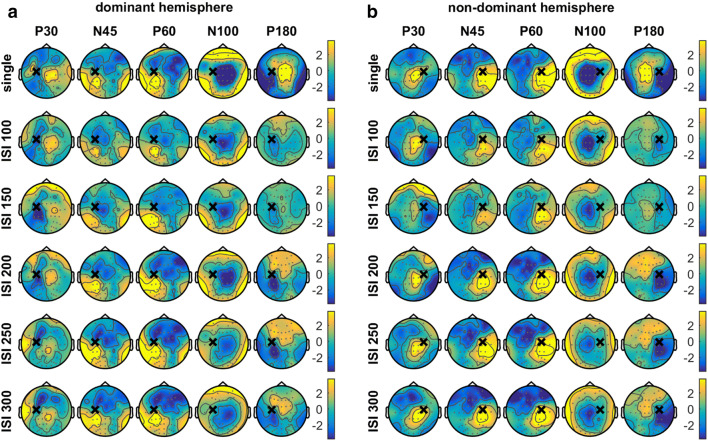
Topographical distribution of the characteristic P30, N45, P60, N100 and P180 components when stimulating the **a** dominant or **b** non-dominant hemisphere during session 1. Spatiotemporal dynamics was comparable for single and paired pulse TMS, and stimulation of the dominant and non-dominant hemispheres resulted in a mirrored pattern. The black cross indicates the stimulation site and the grey dots the 64 electrode positions. Yellow areas indicate a positive amplitude and blue areas a negative amplitude

The average single pulse TEP contained all the characteristic TEP components at electrode Cz for both stimulated hemispheres, see top panels Fig. 2. These P30, N45, P60, N100 and P180 components were also visible in the average paired pulse TEP for ISIs between 100 and 300 ms, see top panels Fig. 3. The topographical distribution of each TEP component was comparable for single and paired pulse TMS, and showed a mirrored spatiotemporal dynamics for stimulation of the dominant and non-dominant hemispheres, see Fig. 4. The P30 component is mainly expressed centrally, the N45 more frontal, the P60 near the stimulation site, the N100 centrally and the P180 component in centrofrontal areas.

### Stability of the Single and Paired Pulse TEP

Time had no significant effect on the single pulse TEP, see Fig. 2. Also, the paired pulse TEP did not differ significantly between both TMS sessions. No significant clusters were found for stimulation of the dominant and non-dominant hemispheres when comparing TEPs from both TMS sessions. In addition, no significant effect of coil positioning method was found when comparing TEPs obtained by manual and robot-guided positioning: no significant clusters for both stimulated hemispheres and TMS sessions. Although we found no effect of stimulated hemisphere during the first TMS session, the paired pulse TEPs measured after stimulating the dominant and non-dominant hemispheres differed twice significantly during session 2. One significant positive cluster (*p* = 0.0047, *d* = 1.1) was found for ISI 100 ms and one significant negative cluster (*p* < 0.001, *d* = − 1.3) for ISI 150 ms. Correlating the N100 and P180 amplitudes of the single and paired pulse TEPs of all subjects measured during the first and second session showed a moderate repeatability, except for ISI 100 ms were repeatability was poor (ICC single pulse: N100 = 0.63, P180 = 0.75; ICC ISI 100 ms: N100 = 0.49, P180 = 0.37; ICC ISI 150 ms: N100 = 0.61, P180 = 0.65; ICC ISI 200 ms: N100 = 0.67, P180 = 0.65; ICC ISI 250 ms: N100 = 0.66, P180 = 0.60; ICC ISI 300 ms: N100 = 0.64, P180 = 0.74).

### LICI of the Paired Pulse TEP

**Fig. 5:**
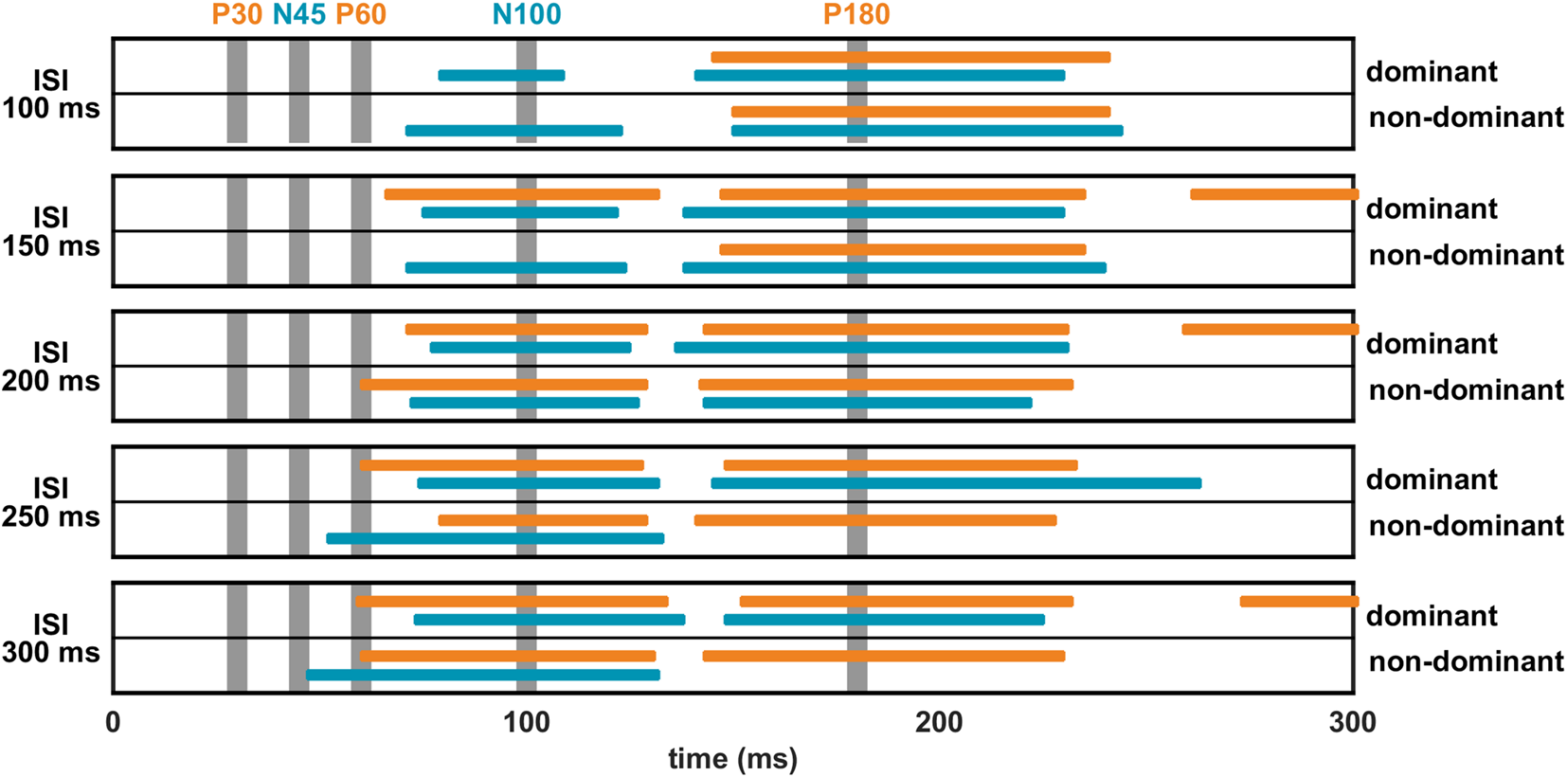
Overview of all significant LICI clusters (single versus paired pulse TEP) found when stimulating the dominant and non-dominant hemispheres during session 1. Positive clusters are presented in orange indicating a reduction of positive amplitudes and negative clusters in blue indicating a reduction of negative amplitudes. Besides the negative N100 and positive P180 clusters, also significant positive N100 and negative P180 clusters were found, as well as positive late clusters. The vertical grey bars indicate the time corresponding to the P30, N45, P60, N100 and P180 components

Since we found moderate repeatability and no significant effect of time on both the single and paired pulse TEP, only LICI outcomes of session 1 are evaluated and presented. Compared to the single pulse TEP at electrode Cz, we found strong suppression of the late paired pulse TEP components (N100 and P180), while the early components (P30, N45 and P60) remained unaffected, see Figs. 1c and 3. Assessment of LICI (single versus paired pulse TEP) showed a significant negative N100 and positive P180 cluster at all ISIs between 100 and 300 ms for stimulation of both the dominant and non-dominant hemisphere (negative N100 clusters: *p* ≤ 0.005, range *d*: − 1.0 to − 1.5; positive P180 clusters: *p* ≤ 0.001, range *d*: 1.0 to 1.8). However, no significant clusters were found for the P30, N45 and P60 components, see Fig. 3. Topographical LICI plots showed significant reduction of the N100 and P180 for the central areas, corresponding to the expression site of these components. In addition to the negative N100 and positive P180 clusters, we often also found a significant positive N100 (*p* ≤ 0.01, range *d*: 0.9 to 1.6) and negative P180 cluster (*p* ≤ 0.003, range *d*: − 1.1 to − 2.1), as well as a late positive cluster (*p* ≤ 0.01, range *d*: 1.0 to 1.3) around 280 ms, see Fig. 5. The positive N100 and negative P180 clusters were located bilaterally in occipito-temporal areas and the late positive cluster occipitally on the contralateral side. For a complete overview of the spatiotemporal dynamics of LICI, please see the movie (Online Resource 1).

## Discussion

In this study we evaluated the spatiotemporal dynamics and stability of the single and paired pulse TEP. The topographical distribution of the P30, N45, P60, N100 and P180 components was comparable for single and paired pulse TMS. Stimulation of the dominant and non-dominant hemispheres resulted in a mirrored spatiotemporal dynamics. TMS-EEG outcomes appear to be stable, as single and paired pulse TEPs were not significantly affected by either stimulated hemisphere, time or coil positioning method. Furthermore, moderate repeatability was found for the most prominent N100 and P180 components. In addition, we assessed LICI at the cortical level for ISIs between 100 and 300 ms by comparing the single versus paired pulse TEP. For all ISIs, it was characterized by significant suppression of the late N100 and P180 components in the central areas, without affecting the early P30, N45 and P60 components.

A reduction of the N100 and P180 components was found consistently for all five ISIs in the range 100–300 ms. Accordingly, Opie et al. (2017) found no significant differences in LICI expression for ISIs 100 and 150 ms (Opie et al. 2017). TEP modulation was also largely identical for SICI (ISI 2 ms) and LICI (ISI 100 ms) (Premoli et al. 2018). These findings seem to contradict with pharmaco-TMS-EMG studies showing two distinct inhibitory mechanisms. SICI is associated with GABA-A receptor mediated inhibition (Kujirai et al. 1993; Di Lazzaro et al. 2007), while LICI reflects GABA-B receptor mediated inhibition (McDonnell et al. 2006; Müller-Dahlhaus et al. 2008). Thus, it appears that knowledge obtained with the TMS-EMG paradigm is not directly translatable to SICI and LICI measured with TMS-EEG (Opie et al. 2017; Premoli et al. 2018). Single pulse pharmaco-TMS-EEG studies demonstrated that the N45 and N100 components are related to GABA-A and GABA-B receptor mediated inhibition, respectively (Premoli et al. 2014a). The fact that we found suppression of the N100 component when assessing LICI, points more to GABA-B than GABA-A receptor mediated inhibition. Future pharmaco-TMS-EEG studies, including a wider range of ISIs, are needed to further unravel the inhibitory mechanisms underlying LICI at the cortical level.

We found that only the late N100 and P180 components were suppressed, which corresponds with recent findings for SICI (Premoli et al. 2018). This is at variance with previous findings of suppression of both early and late TEP components at ISI 100 ms (Premoli et al. 2014b; Opie et al. 2017, 2018), and a significant decrease of the N40, N100 and P180 components at ISI 150 ms, but not the P30 (Opie et al. 2017, 2018). Discrepancies might be partly due to differences in stimulation intensity. Rogasch et al. (2013) showed that increasing the test pulse intensity results in decreased LICI of the early P30 and P60 components, without affecting later components (Rogasch et al. 2013). The fact that we stimulated at an intensity of 120% rMT, instead of 100% rMT like Premoli et al. (2014b), could explain that no significant clusters were found for the P30, N45 and P60 components. Furthermore, we only applied fifty single and paired pulses which might be insufficient to measure early TEP components reliably, as, due to their smaller amplitude, the signal-to-noise ratio is relatively smaller. In addition, inconsistencies might have occurred because we evaluated the entire TEP response over a period of 300 ms, instead of focusing on shorter periods around the TEP components.

The majority of significant clusters could be assigned to one of the characteristic TEP components, suggesting that they contain most of the essential information regarding LICI. However, when stimulating the dominant hemisphere, we additionally found a late positive cluster around 280 ms for ISIs 150, 200 and 300 ms. Although the relevance of this late cluster remains speculative, source localization techniques may assist in elucidating its origin. Furthermore, we recommend that analysis of TMS-EEG data includes evaluation of the late TEP response, as single pulse TMS-EEG studies showed the potential of late responses as a biomarker for epilepsy (Valentin et al. 2008; Shafi et al. 2015). Source localization can also help to gain insight into the presence and distribution of dipoles. Just as Opie et al. (2017, 2018), we often found positive N100 and negative P180 clusters in occipito-temporal areas in addition to the central clusters, indicating an underlying dipole (Opie et al. 2017, 2018).

Before evaluating the paired pulse TEP, we applied a method to correct for the influence of late conditioning response components on early test response components (Daskalakis et al. 2008; Premoli et al. 2014b). However, this method did not take into account the likely modulation of late conditioned response components caused by giving the test pulse (Premoli et al. 2014b). The fact that it is not possible to correct for both interactions due to their unknown entanglement, makes this paired pulse correction method suboptimal. As an alternative, our study suggests that to estimate LICI at the cortical level, stimulation at ISI 300 ms can reliably be used instead of ISI 100 ms as LICI expression was comparable for both ISIs, just as the topographical distribution of TEP components. More importantly, where the conditioning pulse TEP is still ongoing when applying the test pulse after 100 ms, EEG activity has visually returned to baseline at 300 ms, see Fig. 1. This implies that paired pulse stimulation at ISI 300 ms allows assessment of LICI, without the need of using any correction method. Indeed, we also found strong suppression of the N100 and P180 components, without affecting the early P30, N45 and P60 components (results not shown), for LICI at ISI 300 ms when no correction method was applied. Additionally, paired pulse TEPs at ISI 300 were still not significantly affected by either stimulated hemisphere, time or coil positioning method (results not shown).

Clinical applicability of TMS-EEG, as well as the ability to use our findings from healthy subjects as reference values for future TMS-EEG studies, depend on the stability and repeatability over time of the TEP. Our findings indicate the stability of the single and paired pulse TEP in a rather heterogeneous group of healthy subjects. In line with previous studies, spatiotemporal dynamics of the paired pulse TEP showed great resemblance between ISIs (Opie et al. 2017, 2018) and was comparable to the single pulse TEP (Paus et al. 2001; Bonato et al. 2006; Ferreri et al. 2011; Premoli et al. 2014a). Stimulation of the dominant and non-dominant hemisphere resulted in a mirrored pattern without significantly affecting the TEPs, except for ISIs 100 and 150 ms during session 2. In addition, we found no significant effect of time on the single and paired pulse TEPs and a moderate repeatability (ICC > 0.6) for the most prominent N100 and P180 components, except for a poor repeatability at ISI 100 ms. Previous studies showed a high reproducibility of both single (Lioumis et al. 2009; Casarotto et al. 2010) and paired pulse TEPs after one week (Farzan et al. 2010; Premoli et al. 2014b) and a good repeatability of the single pulse TEP during the day (ter Braack et al. 2018). Furthermore, manual coil positioning seems sufficient given the limited added value of robot-guided positioning. In agreement with our earlier TMS-EMG findings on a group level (de Goede and van Putten 2017), no significant effect was found for stimulated hemisphere, time and coil positioning method pointing towards the stability of TMS-EEG.

### Limitations

This study is limited by the fact that we only evaluated the effect of stimulated hemisphere, time and coil positioning method. However, there are more factors that need to be investigated because they may influence the single and paired pulse TEP, like pulse waveform, stimulation intensity, age, mental state or coil orientation and tilt. To evaluate stability of the single and paired pulse TEPs we used the cluster-based permutation analysis to test for differences between two conditions (dominant versus non-dominant hemisphere, session 1 versus session 2 and manual versus robot-guided coil positioning). Not finding significant differences points towards a stable measure, although *t*-tests are designed to test for differences rather than similarities between conditions. In addition, we used the ICC to estimate the absolute agreement between repeated sessions, which is a more appropriate measure to assess repeatability (Shrout and Fleiss 1979; Kerwin et al. 2018). However, where it is still possible to include all spatiotemporal information of the multidimensional TMS-EEG data in the cluster-based permutation analysis (Maris and Oostenveld 2007), this is not the case for ICC. Here a specific electrode or region of interest (ROI) and a specific timepoint are selected, leaving aside a major part of the collected data. As shown by Kerwin et al. (2018), the choice of ROI and timepoint could largely influence the test-retest reliability (Kerwin et al. 2018). The ability to include all spatiotemporal information in the ICC analysis, would improve repeatability testing of single and paired pulse TEPs.

Another limitation is that we applied single trial PCA to reduce the first large TMS artifact and second muscle artifact in our EEG data. PCA has shown to be an effective method to reduce both artifacts simultaneously (ter Braack et al. 2013a; Rogasch et al. 2017). Since PCA might not be the most optimal method to remove high-amplitude and high-frequency artifacts, another commonly used procedure is interpolation of the TMS artifact followed by independent component analysis (ICA) or PCA to remove residual artifacts. EEG data can be contaminated by other artifacts as well, such as eye-blinks, auditory and somatosensory artifacts (Ilmoniemi and Kičić 2010; Ilmoniemi et al. 2015). Currently there is no generally accepted method for artifact removal. However, a combination of multiple methods, including ICA or PCA, seems more appropriate to remove all types of artifacts without significantly affecting the TEP. Even though we strongly encourage the recent development of (fully) automated artifact rejection algorithms (Atluri et al. 2016; Rogasch et al. 2017; Wu et al. 2018), they are mainly tested on the single pulse TEP. More research is needed to make these algorithms suitable for paired pulse TMS-EEG data. In addition, we always removed the first four principal components, while it may be better to manually determine and adjust this number per subject (ter Braack et al. 2013a).

We first applied fifty single pulses, followed by the paired pulses in a randomized order. This may have influenced our LICI findings, as changes occurred during the TMS session (e.g. coil movement away from the hotspot or slow change in attention level) could have affected the single and paired pulse TEPs differently. It would have been more optimal to also randomize the single pulses with the paired pulses.

Even though subjects listened to adaptive noise to mask the sound of the TMS pulses, TEPs are most likely still influenced by auditory input. In particular, the late N100 and P180 components are strongly correlated to auditory input (Tiitinen et al. 1999; Nikouline et al. 1999). Since LICI was characterized by suppression of these late components, we cannot rule out the possibility that our observations may (partially) result from differences in auditory and somatosensory sensations experienced by subjects for single and paired pulse TMS. Due to the absence of a control condition, such as sham stimulation, we cannot reliably assess the potential contribution of remaining auditory processing (despite sound masking) and somatosensory input from the skin or skull. Recently, Conde et al. (2019) applied sham stimulation to the frontal and parietal cortex, mimicking the auditory and somatosensory sensations evoked by real TMS. They showed substantial similarities between real TMS and sham evoked EEG responses (Conde et al. 2019), stressing the need for including a reliable sham condition in future TMS-EEG studies.

## Conclusions

LICI at the cortical level was characterized by significant suppression of the late N100 and P180 components, without affecting the early P30, N45 and P60 components. As LICI expression showed great resemblance between ISIs, stimulation at ISI 300 ms is preferred because paired pulse correction seems superfluous here. Despite strong suppression at the expression site of the late TEP components, the topographical distribution of the characteristic components was comparable for the single and paired pulse TEP. These spatiotemporal dynamics in healthy subjects can serve as reference values for future neuropsychiatric and pharmacological studies, as cortical excitability is modified for various central nervous system conditions and central acting drugs. We found no significant effect of stimulated hemisphere, time and coil positioning method and a moderate repeatability over time, indicating the stability of TMS-EEG outcomes as a potential clinical biomarker.

## Electronic supplementary material

Below is the link to the electronic supplementary material.

Overview of spatiotemporal dynamics of LICI for all five ISIs over 300 ms. Topographical LICI expression when stimulating the dominant (top row) or non-dominant hemisphere (bottom row) for session 1. The late N100 and P180 components are strongly suppressed, while the early P30, N45 and P60 components remained unaffected. Besides the negative N100 and positive P180 clusters, also significant positive N100 and negative P180 clusters are found, as well as positive late clusters. The black cross indicates the stimulation site, the grey dots the 64 electrodes, and the red dots electrodes that show a significant difference between single and paired pulse TEPs. Yellow areas indicate a reduction of positive amplitudes and blue a reduction of negative amplitudes. Supplementary material 1 (MP4 16833.2 kb)
